# Supplementary material for: Heat adaptation of phage T7 under an extended genetic code
Source: Virus Evol. 2021 Dec 1;7(2):veab100. doi: 10.1093/ve/veab100 (PMC8923235; doi:10.1093/ve/veab100)
Supplement: veab100_Supp [file veab100_supp.zip › Sequencing_Analysis.zip!/Sequencing_Analysis/A2/evidence/JC_SIDE_1_48.html]

BRESEQ :: Evidence


| New junction evidence | | | | | | | | | | | |
| --- | --- | --- | --- | --- | --- | --- | --- | --- | --- | --- | --- |
|  | | seq id | position | reads (cov) | reads (cov) | score | skew | freq | annotation | gene | product |
| \* | ? | NC\_001604.1 | = 8253 | 443 (0.970) | 7 (0.020) | 7/584 | 20.4 | 1.4% | intergenic (–/–) | *–/–* | –/– |
|  | ? | NC\_001604.1 | 8503 = | 536 (1.210) |  |  |  |  | intergenic (–/–) | *–/–* | –/– |
| Rejected: Coverage evenness skew score above cutoff. | | | | | | | | | | | |
| Rejected: Frequency below/above cutoff threshold. | | | | | | | | | | | |

Only 100 of 446 total aligned reads displayed.  

`CCGTGCTGACCGTTCAATCGTATGTGCGAGCGAGCGCCGAGCTAAGATACCTCTTATTGGTAACACAGTTCCTTTGGCACCGAGCGTCCACATCATTATCACCCGTGGTGACTTTGAGAAAGCAATAGACAAGAAACGTCCGGTTCTTAGTGTGGCAGTGACCCGCTTCCCGTTCGTCCGTCTGTTACTCAAACGAATCAAGGAGGTGTTCTGATGGGACTGTTAGATGGTGAAGCCTGGGAAAAAGAAAACCCGCCAGTACAAGCAACTGGGTGTATAGCTTGCTTAGAGAAAGATGACCGTTATCCACACACCTGTAACAAAGGAGCTAACGATATGACCGAACGTGAACAAGAGATGATCATTAAGTTGATAGACAATAATGAAGGTCGCCCAGATGATTTGAATGGCTGCGGTATTCTCTGCTCCAATGTCCCTTGCCACCTCTGCCCCGCAAATAACGATCAAAAGATAACCTTAGGTGAAATCCGAGCGATGGACCCACGTAAACCACATCTGAATAAACCTGAGGTAACTCCTACAGATGACCAGCCTTCCGCTGAGACAATCGAAGGTGTCACTAAGCCTTCCCACTACATGC  >  NC_001604.1/7953‑8553`  

                                                                                                                                                                                                                                                                                                            `|`                                                                                                                                                                                                                                                                                                              
`ccGTGCTGACCGTTCAATCGTATGTGCGAGCGAGCGCCGAGCTAAGATACCTCTTATTGGTAACACAGTTCCTTTGGCACCGAGCGTCCACATCATTCTCACCCTTGGTGACTTTGAGAAAGCAATCGACAAGACACGTCCGTTTCTTAGTGTGGCAGTGACCCGCTTCCCGTTCGTCCGTCTGTTACTCAACCGAATCAAGGAGGTGTTCTGTTGGTCCTGTTAGTTGGTGAAGCCTGTGAAAAAGAAAACCCGCCAGTACAAGCACCTGGTTGTATAGCTTGCTTAGAGAAAGATGAcc                                                                                                                                                                                                                                                                                                              >  1:564737/1‑301 (MQ=255)`  
  `gTGCTTAACGTTAAATGGGATGTGAGAGAGAGAGGAGAGATAAGATACCTCTTATTGGTAACAAAGTTCCTTTGGCATCGAGCGTACACAGCATGATCAACCGTGGTGACTTTGAGAAAGCAATAGACAAGAACCGTCCGGTTCTTAGTGTGGCAGTGACCCGCTTCCCGTTCGTCCGTCTGTTACTCAAACGAATCAAGGAGGTGTTCTGATGGGACTGTTAGATGGTGAAGCCTGTGAAAAAGAAAACCAGCCAGTACAAGCAACTGGGTGTATAGCTTGCTTAGAGAAAGATGACAGt                                                                                                                                                                                                                                                                                                            <  1:783181/301‑1 (MQ=255)`  
      `gtataagagacaggTAGGTGCGAGCGAGCGCCGAGCTAAGATACCTCTTATTGGTAACACAGTTCCTTTGGCACCGAGCGTCCACATCATTATCACACGTGGTGACTTTGAGAAAGCAATAGACAAGAAACGTCCGGTTCTTAGTGTGGCAGTGACCAGCTTCCCGTTCGTCCGTCTGTTACTCAAACGAATCAAGGAGGTGTTCTGATGGGACTGTTAGATGGTGAAGCCTGGGAAAAAGAAAACCCGCCAGTACAAGCAACTGGGTGTATAGCTTGCTTAGAGAAAGATGACCATTATc                                                                                                                                                                                                                                                                                                        <  1:775844/288‑1 (MQ=255)`  
         `ccGTTCAATCGTATGTGCGAGCGAGCGCCGAGCTAAGATACCTCTTATTGGTAACACCGTTCCTTTGGCACCGAGCGTCCACATCATTATCACCCGTGGTGACTTTTAGAAAGCAATAGACAAGAAACGTCCGGTTCTTAGTGTGGCAGTGATCCGCTTCCCGTTCGTCCGTCTGTTACTCAAACGCATCAAGGAGGTGTTCTGATGGGACTGTTATATGGTGAAGCCTGGGTAAAAGAAACCCCGCCAGTACAAGCAACTGGGTGTCTAGCTTGCTTAGAGAACGTTGACCCTTATCcac                                                                                                                                                                                                                                                                                                     >  1:546518/1‑301 (MQ=255)`  
           `gTTCAATCGTATGTGCGGGCGAGCGCCGAGCTAAGATACCTATTATTGGTAACACAGTTCCTTTGGCACCGAGCGTCCACATCATTATCACCAGTGGTGACTTTGAGAAAGCAATAGACAAGAAACGTCCGGTTCTTAGTGTGGCAGTGACCCGCTTCCCGTTCGTCCGTCTGTTACTCAAACGAATCAAGGAGGTGTTCTGATGGGACTGTTAGATGGTGAAGCCTGGAAAAAAGAAAACCCGCCAGTACAAGCAACTGGGTGTATAGCTTGCTTAGAGAAAGATGACCGTTATCcacac                                                                                                                                                                                                                                                                                                   <  1:272167/301‑1 (MQ=255)`  
           `gTTCAATCGTATGTGCGAGCGAGCGCCGAGCTAAGATACCTCTTATTGGTAACACAGTTCCTTTGGCACCGAGCGTCCACATCATTATCACCCGTAGTGACTTTGAGAAAGCAATAGACAAGAAACGTCCGGTTCTTGGTGTGGCAGTGACCCGCTTCCCGTTCGTCCGTCTGTTACTCAAACGAATAAAGGAGGTGTTCTGATGGGACTGTTAGATGGTGAAGCCTGGGAAAAAGAAAACCCGCCAGTACAAGCAACTGGGTGTATAGCTTGCTTAGAGAAAGATGACCGTTATCcacac                                                                                                                                                                                                                                                                                                   >  1:325251/1‑301 (MQ=255)`  
           `aagagacagTATGTGTAAGCGAGCGCCGAGCTAAGATACCTCTTATTGGTAACACAGTTCCTTTGGCACCGAGCGTCCACATCATTATCACCCGTGGTGACTTTGAGAAAGCAATAGACAAGAAACGTCCGGTTCTTAGTGTGGCAGTGACCCGCTTCCCGTTCGTCCGTCTGTTACTCAAACGAATCAAGGAGGTGTTCTGATGGGACTGTTAGATTGTGAAGCCTGGGAAAAAGAAAACCCGCCAGTACAAGCAACTGGGTGTATAGCTTGCTTAGAGAAATATGACCGTTATCcacac                                                                                                                                                                                                                                                                                                   <  1:197783/293‑1 (MQ=255)`  
             `tccaTCGTATGTGCGTGCGCGCGCCGGGCTAAGATTCCTCTTATTGGTAACACAATTCCTTTGGCCCCGAGCTTCCACATCTTTTTCCCCCGTTGTGTCTTTGAGAAAGCATTAGACCAGAAACGTCCGGTTCTTAGTGTGGCAGTTACCCGCTTCCCGTTCGTCCGTCTGTTACTCAAACGGATCAAGGAGGTGTTCTGATTGGACTGTTAGATGGTGAAGCCTTGGAAAAAGAAAACCCGCCAGTACAAGCAACTGGGTGTATAGCTTGCTTAGAGAAAGATGACCGTTATCCacacac                                                                                                                                                                                                                                                                                                 <  1:66401/298‑1 (MQ=255)`  
             `tCAATCGTATGTGGGAGGGAGGGCCGAGCTAAGATACCTCTTATTGGTAACACAGTTCCTTTGGCACCGAGCGTCCACATCATTATCACCCGTGGTGACTTTGAGAAAGCAATAGACAAGAAACGTCCGGTTCTTAGTGTGGCAGTGACCCGCTTCCCGTTCGTCCGTCTGTTACTCAAACGAATCAAGGAGGTGTTCTGATGGGACTGTTAGATGGTGAATCCTGGGAAAAAGAAAACCCGCCAGTACAAGCAACTGGGTGTATAGCTTGCTTAGAGAAAGATGACCGTTATCCacacac                                                                                                                                                                                                                                                                                                 <  1:202548/301‑1 (MQ=255)`  
             `tCAATCGTAGGTGAGAGGGTGCGACGGGCTAATATACCTCTTATTAGTAACACAGTGCCTTTGGCACCGGTCGTCAACATCATTATCACCCGTGGTGACTTTGAGAAAGCAATAGACAAGAAACGTCCGGTTCGTAGTGTGGCAGTGACCCGCTTCCCTTTCGTCCGTCTGTTACTCAAACGATTCAAGGAGGTGTTCTGATTGGACTGTTAGATGGTGAAGCCTGGGAAAAAGAAAACCCGCCAGTACAAGCAACTGGGTGTATAGCTTGCTTAGAGAAAGATGACCGTTATCCacacac                                                                                                                                                                                                                                                                                                 <  1:823532/301‑1 (MQ=255)`  
              `cAATCTTATGTGCGAGCGAGCGCCGAGCTAAGATACCTCTTATTGGTAACACAGTTCCTTTGGCACCGAGCGTCCACATCATTAGCACCCGTGGTGACTTTGAGAAAGCAATAGACAAGAAACGTCCGGTTCTTAGTGTGGCAGTGACCCGCTTCCCGTTCGTCCGTCTGTTACTCAAACGAATCAAGGAGGTGTTCTGATGGGACTGTTAGATTGTGAAGCCTGGGAAAAAGAAAACCCGCCAGTACAAGCAACTGGGTGTATAGCTTGCTTAGAGAAAGATGACCGTTATCCACACAcc                                                                                                                                                                                                                                                                                                <  1:361542/301‑1 (MQ=255)`  
              `agctCGTATGTGCGAGCGAGCTCCTAGCTAAGATACCTCTTATTGGTAACACAGTTCCTTTGGCACCGGGCGTCCACATCATTATCACCCGTGGTGACTTTGAGAAAGCAATAGACAAGAAACGTCCGGTTCTTAGTGTGGCAGTGACCCGCTTCCCGTTCGTCCGTCTGTTACTCAAACGAATCAAGGAGGTGTTCTGATGGGACTGTTAGATGGTGAAGCCTGGGAAAAAGAAAACCCGCCAGTACAAGCAACTGGGTGTATAGCTTGCTTAGAGAAAGATGACCGTTATCCACACAcc                                                                                                                                                                                                                                                                                                <  1:674341/298‑1 (MQ=255)`  
                `aTCGTATGTGCGAGCGAGCTCCGAGCTAAGATACCTCTTATTGGTAACACAGTTCCTTTGGCACCGAGCGTCCACATCATTATCACCCGTGGTGACTTTGAGAAAGCAATAGACAAGAAACGTCCTGTTCTTAGTGTGGCAGTGACCCGCTTCCCGTTCGTCCGTCTGTTACTCAAACGAATCAAGGAGGTGTTCTGATGGGACTGTTAGATGGTGAAGCCTGGGAAAAAGAAAACCCGCCAGTACAAGCAACTGGGTGTATAGCTTGCTTAGAGAAAGATGAccagccttccgctgagac                                                                                                                                                                                                                                                                                              >  1:349271/1‑285 (MQ=255)`  
                   `tgtagaggagtataagagaaaGCTAAGATACCTGGTATTGGTAACAAAGTTACTTTGGTACCTAGCGTCCAATTCATTATCACCCGGGGTTACTGGGAGAAAGAAATAGCCAATAAAGTTCCGGTTTTAAGTGTGGAAGTGACCCGCTTCCCGTTCGTACGTCTGTGACTCAAACGAATCAAGGAGGTGTTCTGATGGGACTGTTAGATGGTGAAGCCTGGGAAAAAGAAAACCCGCCAGTACAAGCAACGGGGTGTATAGCTTGTTGAGAGAAAGATGACCGTTATCCACACACCTGTaa                                                                                                                                                                                                                                                                                           <  1:743575/281‑1 (MQ=255)`  
                   `ggagatgtgaataagagacaggCTAAGATACCTCTTATTGGTAACCCAGTTCCTTTGGAACCGAGCGTCCACATCATTATCACCCGTGGGGACTTTGAGAAAGCAATAGACAAGAAACGTCCGGTTCTTAGTGTGGCAGTGACCCGCTTCCCGTTCGTCCGTCTGTTACTAAAACGAATCAAGGAGGTGTTCTGATGGGACTGTTAGATGGTGAAGCCTGGGAAAAAGAAAACCCGCCAGTACAAGCAACTGGGTGTATAGCTTGCTTAGAGAAAGATGACCGTTATCCACACACCTGTaa                                                                                                                                                                                                                                                                                           <  1:136159/280‑1 (MQ=255)`  
                      `tgtgCCGGCGAGCGCCGAGCTAAGAACCCTCTTATTGGTACCACAGTTCTTTTGGCACCAAGCGTCCACATCATTATCACCCGTGGTGACTTTGAGAAAGCAATAGACAAGAAACGTCCGGTTCTTAGTGTGGCAGTGACCCGCTTCCCGTTCGTCCGTCTATTACTCAAACGAATCAAGGAGGTGTTATGATGGGACTGTTAGATGGTGAAGCCTGGGAAAAAGAAAACCCGCCAGTACAAGCAACTGGGTGTATAGCTTGCTTAGAGAAAGATGACCGTTATCCACACACCTGTAACaa                                                                                                                                                                                                                                                                                        <  1:44733/301‑1 (MQ=255)`  
                        `tgCGAGCGAGCGCCGAGCTAAGATACCTCTTATTGGTAACACAGTTCCTTTGGCACCGAGCGTCCACATCATTATCACCCGTGGTGACTTTGAGAAAGCAATAGACAAGTAACGTCCGGTTCTTAGTGTGGCAGTGACCCGCTTCCCGTTCGTCCGTCTGTTACTCAAACGAATCAAGGAGGTGTTCTGATGGGACTGTTAGATGGTGAAGCCTGGGAAAAAGAAAACCCGCCAGTACAAGCAACTGGGTGTATAGCTTGCTTAGAGAAAGATGAccagccttccgcttagacaatcgaag                                                                                                                                                                                                                                                                                      >  1:248320/1‑277 (MQ=255)`  
                         `gcgggcgaGCGCCGAGCTAAGATCCCTATTATTGGTAACACAGTTCCTTTGGCACCGAGCGTCCACATCATTATCACCCGTGGTGACTTTGAGAAAGCAATAGACAAGAAACGTCCGGTTCTTAGTGTGGCAGTGACCCGCTTCCCGTTCGTCCGTCTGTTACTCAAACGAATCAAGGAGGTGTTCTGATGGGACTGTTAGATGGTGAAGCCTGGGAAAAAGAAAACCCGCCAGTACAAGCAACTGGGTGTATAGCTTGCTTAGAGAAAGATGACCGTTATCCACACACATGTAACAAAgg                                                                                                                                                                                                                                                                                     <  1:624278/301‑1 (MQ=255)`  
                         `ccaagcgagccCCCAGCTAAGACACTTCTAATTGGTAACACATTTCCTTTGCCACCGAGCGTCCACATCCGTATCACCCGTGGTGACTGTTAGAAAGCAATAGACAAGAAACGTCCGGTTCTTAGTTTGGCAGTGACCCGCTGCCCTTTCGTCCGTCTGTTACTCAAACGAATCAAGGAGGTGTTCTGATGGGACTTTTAGATGGTGAAGCCTGGGAAAAAGAAAACCCGCCAGTACAAGCAACTGGGTGTATAGCTTGCTTAGAGAAAGATGACCGTTATCCACACACCTGTAACAAAgg                                                                                                                                                                                                                                                                                     <  1:127817/298‑1 (MQ=255)`  
                                   `gcCGAGCTAAGATACCTCTTATTGGTAACACAGTTCATTTGGCAACGAGCGTCCACAACATTATCACCCGGGGTGAATTTGAGAAAGCAATAGACAAGAAACGTCCGGTTCTTAGTGTGGCAGTGACCCGCTTCCCGTTCGTCCGTCTGTTACTCAAACGAATCAAGGAGGTGTTCTGATGGGACTGTTAGATGGTGAAGCCTGGGAAAAAGAAAACCCGCCAGTACAAGCAACTGGGTGTATAGCTTGCTTAGAGAAAGATGACCGTTATCCACACACCTGTAACAAAGGAGCTAACGat                                                                                                                                                                                                                                                                           <  1:506007/301‑1 (MQ=255)`  
                                      `gAGCTAAGATACCTCTTATTGGTAACACAGTTCCTTTGGCACCGAGCGTCCACATCATTATCACCCGTGGTGACTTTGAGAAAGCAATAGACAAGAAACGTCTGGTTCTTAGTGTGGCAGTGACCCGCTTCCCGTTCGTCCGTCTGTTACTCAAACGAATCAAGGAGGTGTTCTGATGGGACTGTTAGATGGTGAAGCCTGGGAAAAAGAAAACCCGCCAGTACAAGCAACTGGGTGTATATCTTGCTTAGAGAAAGATGACCGTTATCCACACAccctgtctcttatacacatctccgag                                                                                                                                                                                                                                                                        >  1:793671/1‑277 (MQ=255)`  
                                        `gCTAATATCCCTTTTATTGGTAACACGGTTCCTTTGGCACCGACCGTCCACACTATTATCACCCGTGATGACTTTGAGAAAGCAATAGACAAGAAACGTCCGGTTCTTAGTGTGGCAGTGACCCGCTTCCCGTTCGTCCGTCTGTTACTCAAACGAATCAAGGAGGTGTTCTGATGGGACTGTTAGATGGTGAAGCCTGGGAAAAAGAAAACCCGCCAGTACAAGCAACTGGGTGTATAGCTTGCTTAGAGAAAGATGACCGTTATCCACACACCTGTAACAAAGGAGCTAACGATATGAc                                                                                                                                                                                                                                                                      <  1:277029/301‑1 (MQ=255)`  
                                        `gCTAAGATACCTCTTATTGGTAACACAGTTCCTTTGGCACCGAGCGTCCACATCATTATCACCCGTGGTGACTTTGAGAAAGCAATAGACAAGAAACGTCCGGTTCTTAGTGTGGCAGTGACCCGCTTCCCGTTCGTCCGTCTGTTACTCAAACGAATCAAGGAGGTGTTCTGATGGGACTGTTAGATGGTGAAGCCTGGGAAAAAGAAAACCCGCCAGTACAAGCAACTGGGTGTATAGCTTGCTTAGAGAAAGATGACCGTTATCCACACAccctgtctcttatacccatctccgagcc                                                                                                                                                                                                                                                                      >  1:345614/1‑275 (MQ=255)`  
                                          `ggggctcggaaatgtgtataagggacagccTTTGGCACCGAGCGTCCACATAATTATCACCCGTGGTAACTTTGAGAAAGCAATAGACAAGAAACGTCCGGTTCTTAGTGTTTCAGTGACCCGCTTCCCGTTCTTCCGTCTGGTACTCAAACGAATCAAGGAGGTGTTCTGATGGGACTGTTAGATGGTGAAGCCTGGGAAAAAGAAAACCCGCCAGTACAAGCAACTGGGTGTATAGCTTGCTTAGAGAAAGATGACCGTTATCCACACACCTGTAACAAAGGAGCTAACGATATGACCg                                                                                                                                                                                                                                                                    <  1:755939/273‑1 (MQ=255)`  
                                              `ttataagagaccggTAACACAGTTCCTTTGGCACGGAGCGTCCAATTCATTATCACCCGTGGTGACTTTGAGAAAGCAATAGACAGGAAACGTGCGGTTCTTAGTGTGGCAGGGACCCGCTTCCCGTTCGTCCGTCAGTTAAGAAAACGAATCGAGGGGGTGTTCTGATGGGACTGTTAGATGGTGAAGCCTGGGAAAAAGAAAACCCGCCAGTAAAAGCAACTGGGTGTATAGCTTGCTTAGAGAAAGATGACCGTTATCCACACAACTGTAACAAAGGAGCTAACGATATGACCGAACg                                                                                                                                                                                                                                                                <  1:427773/289‑1 (MQ=255)`  
                                              `atgtgtataagagacagcacaGTTCCTTTGGCACCGAGCGTCAACATCATTATCACCCGTGGTGACTTTGAGAAAGCAATAGACAAGAAACGTCCGGTTCTTAGTGTGGCAGTGACCCGCTTCCCGTTCGTCCGTCTGTTACTCAAACGAATCAAGGAGGTGTTCTGATGGGACTGTTAGATGGTGAAGCCTGGGAAAAAGAAAACCCGCCAGTACAAGCAACTGGGTGTATAGCTTGCTTAGAGAAAGATGACCGTTATCCACACACCTGTAACAAAGGAGCTAACGATATGACCAAACg                                                                                                                                                                                                                                                                <  1:101411/284‑1 (MQ=255)`  
                                                 `ccTCTTATTGGTAACACAGTTCCTTTGGCACCGAGCGTCCACATCATTATCACCCGTGGTGACTTTGAGAAAGCAATAGACAAGAAACGTCCGGTTCTTAGTGTGGCAGTGACCCGCTTCCCGTTCGTCCGTCTGTTACTCAAACGAATCAAGGAGGTGTTCTGATGGGACTGTTAGATGGTGAAGCCTGGGAAAAAGAAAACCCGCCAGTACAAGCAACTGGGTGTATAGATTGCTTAGAGAAAGATGACCGTTATCCGCACACCTGTAACactgtctcttatacacatctccgagcccc                                                                                                                                                                                                                                                             >  1:227642/1‑273 (MQ=255)`  
                                                   `tctTATTGGTAACACAGTTCGTTTGGCACCGAGCGTCCACATCATTATCACCCGTGTTGACGTTGAGAAAGCAATAGACAAGAAACATCCGGTTCTTAGTGTGGCAGTGACCCGCTTCACGTTCGTCCGTCTGTTACTCAAACGAATCAAGGAGGTGTTCTGATGGGACTGTTAGATGGTGAAGCCTGGGAAAAAGAAAACCAGCCAGTACAAGCAACTGGGTGTATAGCTTGCTTAGAGAAAGATGACCGTTATCCACACACCTGTAACAAAGGAGCTAACGATATGACCGAACGTGAAc                                                                                                                                                                                                                                                           <  1:47813/301‑1 (MQ=255)`  
                                                      `tATTGGTAACACAGTTCCTTTGGCACCGAGCGTCCACAGCATTATCACCCGTGATGACTTTGAGAAAGCAATAGACAAGAAACGTCCGGTTCTTAGTGTGGAAGTGACCCGCTTCCCGTTCGTCCGGCTGTTACTCAAACGAATCAAGGAGGTGTTCTGATGGGACTGTTAGATGGTGAAGCCTGGGAAAAAGAAAACCAGCCAGTACAAGCAACTGGGTGTATAGCTTGCTTAGAGAAAGATGACCGTTATCCACACACCTGTAACAAAGGAGCTAACGATATGACCGAACGTGAACAag                                                                                                                                                                                                                                                        <  1:735579/301‑1 (MQ=255)`  
                                                         `tGGTAACACAGTTCCTTTGGCACCGAGCGTCCACATCATTATCACCCGTGGTGACTTTGAGAAAGCAATAGACAAGAAACGTCCGGTTCTTAGTGTGGCAGTGACCCGCTTCCCGTTCTTCCGTCTGTTACTCAAACGAATCAAGGAGGTGTTCTGATGGGTCTGTTAGATGCTGAAGCCTGGGAAAAAGAAAACCCGCCAGTACAAGCAACTGGGTGTATAGCTTGCTTATAGAAAGATGACCGTTATCCACACACCTGTAACAAAGGAGCTAACGATATGACCGAACGTGAACaatatt                                                                                                                                                                                                                                                     >  1:659159/1‑297 (MQ=255)`  
                                                         `tGGTAACACAGTTCCTTTGGCACCGAGCGTCCACATCATTATCACCCGTGGTGACTTTGAGAAAGCAATAGACAAGAAACGTCCGGTTCTTAGTGTGGCAGTGACCCGCTTCCCGTTCGTCCGTCTGTTACTCAAACTAATCAAGTAGGTGTTCTGATGGGACTGTTAGATGGTGAAGCCTGGTAAAAAGAAAACCCGCCAGTACAAGCAACTGGGTGTATAGCTTGCTTAGAGAAAGATGACCGTTATCCACACACCTGTAACAAAGGAGCTAACGATATGACCGAACGTGAACAAgaga                                                                                                                                                                                                                                                     >  1:702362/1‑301 (MQ=255)`  
                                                          `ggTAACACAGTTCCTTTGGCACCGAGCGTCCACATCATTATCACCAGTGGTGACTTTGAGAAAGCAATAGACAAGAAACGTCCGGTTCTTAGTGTGGCAGTGACCCGCTTCCCGTTCGTCCGTCTGTTACTCAAACGAATCAAGGAGGTGTTCTGATGGGACTGTTAGATGGTGAAGCCTGGGAAAAAGAAAACCCGCCAGTACAAGCAACTGGGTGTATAGCTTGCTTAGAGAAAGATGACAGTTATCCACACACCTGTAACAAAGGAGCTAACGATATGACCGAACGTGAACAAgagac                                                                                                                                                                                                                                                    >  1:763171/1‑300 (MQ=255)`  
                                                           `gTAACACAGTTCCTTTGGAACCGAGCGTCCACATCATTATCACCCGTGGTGACTTTGAGAAAGCAATAGACAAGAAACGTCCGGTTCTTAGTGTGGCAGTGACCCGCTTCCCGTTCGTCCGTCTGTTACTCAAACGAATCAAGGAGGTGTTCTGATGGGACTGTTAGATGGTGAAGCCTGGGAAAAAGAAAACCAGCCAGTACAAGCAACTGGGTGTATAGCTTGCTTAGAGAAAGATGACCGTTATCCACACACCTGTAACAAAGGAGCTAACGATATGACCgctgtctcttatacacat                                                                                                                                                                                                                                                   >  1:681854/1‑284 (MQ=255)`  
                                                             `agggacagcccTTTGGCCCCGAGGCTCCACACCATTATCACCCGTGGTGACTTTGAGAAAGCAATAGACAAGATACGTCCGGTTCTTAGTGTGGCAGTGACCTGCTTCCCGTTCGTCCGTCTGTTACTCAAACGAATCAAGGAGGTGTTCTGATGTGACTGTTAGATGGTGAAGCCTGGGAAAAAGAAAACCCGCCAGTACAAGCAACTGGGTGTATAGCTTGCTTAGAGAAAGATGACCGTTATCCACACACCTGTAACAAAGGAGCTAACGATATGACCGAACGTGAACAAGAgatgat                                                                                                                                                                                                                                                 <  1:418232/292‑1 (MQ=255)`  
                                                              `acatAGTTCCTTTGGCACCGAGCGTCCACATCATTATCACCCGTGGTGACTTTGAGAAAGCAATAGACAAGAAACGTCCGGTTCTTAGTGTGGCAGTGACCCGCTTCCCGTTCGTCCGTCTGTTACTCAAACGAATCAATGAGGTGTTCTGATTGGACTGTTAGATGGTGAAGCCTGGGAAAAAGAAAACCCGCCAGTACAAGCAACTGGGTGTATAGCTTGCTTAGAGAAAGATGACCGTTATCCACACACCTGTAACAAAGGAGCTAACGATATGACCGAACGTGAACAAGAgatgctg                                                                                                                                                                                                                                                >  1:259331/1‑298 (MQ=255)`  
                                                               `cacaGTTCCTTTGGCACCGAGCGTCCACATCATTATCACCCGTGGTGACTTTGAGAAAGTAATAGACAAGAAACGTCCGGTTCTTAGTGTGGCAGTGACCCGCTTCCCGTTCGTCCGTCTGTTACTCAAACGAATCAAGGAGGTGTTCTGATGGGACTGTTAGATGGTGAAGCCTGGGAAAAAGAAAACCCGCCAGTACAAGCAACTGGGTGTATAGCTTGCTTAGAGAAAGATGACCGTTATCCACACACCTGTAACAAAGGAGCTAACGATATGAcctgtctcttatacacatctccga                                                                                                                                                                                                                                               >  1:645180/1‑279 (MQ=255)`  
                                                                `acaGTTCCTTTGGCACCGAGCGTCCACATCATTATCACCCGTGGTGACTTTGAGAAAGCAATAGACAAGAAACGTCCGGTTCTTAGTGTGGCAGTGACCCGCTTCCCGTTCGTCCGTCTGTTACTCAAACGAATCAAGGAGGTGTTCTGATGGGACTGTTAGATGGTGAAGCCTGGGAAAAAGAAAACCCGCCAGTACAAGCAACTGGGTGTATAGCTTGCTTAGAGAAAGATGACCGTTATCCACACACCTGTAACAAAGGAGCTAACGATATGACCGAACGTGAACAAGAGATGATCAt                                                                                                                                                                                                                                              <  1:392614/301‑1 (MQ=255)`  
                                                                     `tCCTTTGGCACCGAGCGTCCAAATAATTATCACCCGTGGTGACTTTGAGAAAGCAATAGACAAGAAACGTCCGGTTCTTAGTGTGGCAGTGACCCGCTTCCCGTTCGTCCGTCTGTTACTCAAACGAATCAAGGAGGTGTTCTGATGGGACTGTTAGATGGTGAAGCCTGGGAAAAAGAAAACCCGCCAGTACAAGCAACTGGGTGTATAGCTTGCTTAGAGAAAGATGACCGTTATCCACACACCTGTAACAAAGGAGCTAACGATATGACCGAACGTGAACAAGAGATGATCATTAAGt                                                                                                                                                                                                                                         <  1:126133/301‑1 (MQ=255)`  
                                                                      `ccTTTGGCACCGAGCGTCCACATCATTATCACCCGTGGTGACTTTGAGAAAGCAATAGACAATAAACGTCCGGTTCTTAGTGTGGCAGTGACCTGCTTCCCGTTCGTCCGTCTGTTACTCAAACGAATCAAGGAGGTGTTCTGATGGGACTGTTAGATGGTGAAGCCTGGGAAAAAGAAAACCCGCCAGTACAAGCAACTGGGTGTATAGCTTGCTTAGAGAAAGATTACCGTTATCCACACACCTGTAACAAAGGAGCTAACGATATGACCGAACGTGAAcctgtctcttatacacatct                                                                                                                                                                                                                                        >  1:288409/1‑282 (MQ=255)`  
                                                                       `cTTTGGCACCGAGCGTCCACATCATTATCACCCGTGGTGACTTTGAGAAAGCAATAGACAAGAAACGTCCGGTTCTTAGTGTGGCAGTGACCCGCTTCCCGTTCGTCCGTCTGTTACTCAAACGAATCAAGGAGGTGTTCTGATGGGACTGTTAGATGGTGAAGCCTGGGAAAAAGAAAACCCGCCAGTACAAGCAACTGGGTGTATAGCTTGCTTAGAGAAAGATGACCGTTATCCACACACCTGTCACAAAGGAGCTAACGATATGACCGAACGTGAACACGAGATGATCATTAAGTTg                                                                                                                                                                                                                                       >  1:317855/1‑301 (MQ=255)`  
                                                                             `gggagatgtgtataagagacagcACCCGTGTTGACTTTGAGAAAGCAATAGACAAGAACCTTCCGTTTCTTAGTGTGGCAGTGACCATCTTCCCGTTCGTCCGTCTGTTACTCAAACGAATCAAGGAGGTGTTCTGATGGGACTGTTAGATGGTGAAGCCTGGGAAAAAGAAAACCCGCCAGTACAAGCAACTGGGTGTATAGCTTGCTTAGAGAAAGATGACCGTTATCCACACACCTGTAACAAAGGAGCTAACGATATGACCGAACGTGAACAAGAGATGATCATTAAGTTGATAGAc                                                                                                                                                                                                                                 <  1:714385/279‑1 (MQ=255)`  
                                                                                       `caacaTCATTATCACCCGTGGTGACTTTGAGAAAGCAATAGACAAGAAACGTCCGGTTCTTAGTGTGGCAGTGACCCGCTTCCCGTTCGTCCGTCTGTTACTCAAACGAATCAAGGAGGTGTTCTGATGGGACTGTTAGATGGTTAAGCCTGGGAAAAAGAAAACCCGCCAGTACAAGCAACTGGGTGTATAGCTTGCTTAGAGAAAGATGACCGTTATCCACATACCTGTAACAAAGGAGCTAACGATATGACCGAACGTGAACAAGAGATGATCATTAAGTTGATAGACAATAATGAAg                                                                                                                                                                                                                       <  1:161414/299‑1 (MQ=255)`  
                                                                                         `acaTCATTATCACCCGTGGTGACTTTGAGAAAGCAATAGACAAGAAACGTCCGGTTCTTAGTGTGGCAGTGACCCGCTTCCCGTTCGTCCGTCTGTTACTCAAACGAATCAAGGAAGTGTTCTGATGGGACTGTTAGATGGTGAAGCCTGGGAAAAAGAAAACCCGCCAGTACAAGCAACTGGGTGTATAGCTTGCTTAGAGAAAGATGACCGTTATCCACACACCTGTAACAAAGGAGCTAACGATATGACCGAACGTGAACAAGAGATGATCATTAAGTTGATAGAcctgtctcttata                                                                                                                                                                                                                     >  1:569490/1‑289 (MQ=255)`  
                                                                                              `atTGTCACCCGTGGTGACTTTGAGAAAGCAATAGACAAGAAACGTCCGGTTCTTAGTGTGGCAGTGACCCGCTTCCCGTTCGTCCGTCTGTTACTCAAACAAATCAAGGAGGTGTTCGGATGGGACTGTTAGATGGTGAAGCCTGGGAAAAAGAAAACCCGCCAGTACAAGCAACTGGGTGTATAGCTTGCTTAGAGAAAGATGACCGTTATCCACACACCTGTAACAAAGGAGCTAACGATATGACCGAACGTGAACAAGAGATGATCATTAAGTTGATAGACAATAATGAAGGtagacc                                                                                                                                                                                                                >  1:584082/1‑296 (MQ=255)`  
                                                                                                `tATCACCCGTGGTGACTTTGAGAAAGCAATAGACAAGAAACGTCCGGTTCTTAGTGTGGCAGTGACCCTCTTCCCGTTCGTCCGTCTGTTACTCAAATGAATCAAGGAGGTGTTCTGATGGGACTGTTAGATGGTGAAGCCCGGG‑AAAAGGAAACCCGCCAGTACAAGCAACTGGGTGTATAGCTTGCTTAGAGAAAGATGACCGTTATCCACACACCTGTAACAAAGGAGCTAACGATATGACCGAACGTGAACAAGAGATGATCATTAAGtctttctcttatacacatctcccagccca                                                                                                                                                                                                             >  1:488909/1‑273 (MQ=255)`  
                                                                                                   `ggggctcggagatgtgtataagagacaggACAAGAAACGTCCGGTTCTTAGTGTGGCAGTGACCCGCTTCCCGTTCGTCCGTCTGTTACTCAAACGAATCAAGGAGGTGTTCTGATGGGACTGTTAGATGGTGAAGCCTGGGAAAAAGAAAACCCGCCAGTACAAGCAACTGGGTGTATAGCTTGCTTAGAGAAAGATGACCGTTATCCACACACCTGTAACAAAGGAGCTAACGATATGACCGAACGTGAACAAGAGATGATCATTAAGTTGATAGACAATAATGAAGGTCGCCCAgatg                                                                                                                                                                                                           <  1:818478/273‑1 (MQ=255)`  
                                                                                                   `cACCCGTGGTGACTTTGAGAAAGCAATAGACAAGAAACGTCCGGTTCTCAGTGTGGCAGTGACCCGCTTCCCGTTCGTCCGTCTGTTACTCAAACGAATCAAGGAGGTGTTCTGATTGGACTGTTAGATGGTGAAGCCTGGGAAAAAGAAAACCCGCCAGTACAAGCAACTGGGTGTATAGCTTGCTTAGAGAAAGATGACCGTTATCCACACACCTGTAACAAAGGAGCTAACGATATGACCGAACGTGAACAAGAGATGATCATTAAGTTGATAGACAATAATGAAGGTCTCCCAgatg                                                                                                                                                                                                           >  1:576112/1‑301 (MQ=255)`  
                                                                                                     `cccGTGGTGACTTTGAGAAAGCAATAGACAAGAAACGTCCGGTTCTTAGTGTGGCAGTGACCCGCTTCCCGTTCGTCCGTCTGTTACTCAAACGAATCAAGGAGGTGTTCTGATGGGACTGTTAGATGGTGAAGCCTGGGAAAAAGAAAACCCGCCAGTACAAGCAACTGGGTGTATAGCTTGCTTAGAGAAAGATGACCGTTATCCACACACCTGTAACAAAGGAGCTAACGATATGACCGAACGTGAACAAGAGATGATCAGTAAGTTGATAGACAATAATGAAGGTCGCCCAgatgat                                                                                                                                                                                                         >  1:469222/1‑301 (MQ=255)`  
                                                                                                     `cccGTGGTGACTTTGAGAAAGCAATAGACAAGAAACGTCCGGTTCTTAGTGTGGCAGTGACCCGCTTCCCGTTCGTCCGTCTGTTACTCAAACGAATCAAGGAGGTGTTCTGATGGGACTGTTAGATGGTGAAGCCTGGGAAAAAGAAAACCCGCCAGTACAAGCAACTGGGTGTATAGCTTGCTTAGAGAAAGATGACCGTTATCCACACACATGTAACAAAGGAGCTAACGATATGACCGAACGTGAACAAGAGATGATCATTAAGTTGATAGACAATAATGactgtctcttatacaca                                                                                                                                                                                                         >  1:463877/1‑285 (MQ=255)`  
                                                                                                           `gttaCTTTGAGAAAGCAATAGACAAGAAACGTCCGGTTCTTAGTGTGGCAGTGACCCGCTTCCCGTTCGTCCGTCTGTTACTCAAACGAATCAAGGAGGTGTTCTGATGGGACTGTTAGATGGTGAAGCCTGGGAAAAATAAAACCCGCCAGTACAAGCAACTGGGTGTATAGCTTGCTTAGAGAAAGATGACCGTTATCCACACACCTGTAACAAAGGAGCTAACGATATGACCTAACGTGAACAAGAGATGATCATTAAGTTGATAGACAATAATGAAGGTCGCCCAGATGATTTGAAt                                                                                                                                                                                                   <  1:634672/298‑1 (MQ=255)`  
                                                                                                                     `agaaGCAATAGACAAGAAACGTCCGGTTCTTAGTGTGGCAGTGACCCGCTTCCCGGTCGTCCGTCTGTTACGCAAACGAATCAAGGAGGTGTTCTGATGGGACTGTTAGATGGTGAAGCCTGGGAAAAAGAAAACCCGCCAGTACAAGCAACTGGGTGTATAGCTTGCTTAGAGAAAGATGACCGTTATCCACACACCTGTAACAAAGGAGCTAACGATATTACCGAACGTGAACAAGAGATGATCATTAAGTTGATAGACAATAATGAAGGTCGCCCAGATGATTTGAATGGCTGCGGTa                                                                                                                                                                                         <  1:739184/299‑1 (MQ=255)`  
                                                                                                                          `tgtataagagacagcGTCCGTTTCTTAGGGTGGCAGGGACCCGCTTCCCGTTCGTCCTGCTGTTACTCAAACGAATCAAGGAGGTGTTCTGATGGGACTGTTAGAGGGTGAAGCCTGGGAAAAAGAAAACCCGCCAGTACAAGCAACTGGGTGTATAGCTTGCTTAGAGAAAGATGACTGTTATCCACACACCTGTAACAAAGGAGCTAACGATATGACCGAACGTGAACAAGAGATGATCATTAAGTTGATAGACAATAATGAAGGTCGCCCAGATGATTTGAATGGCTGCGGTATtctc                                                                                                                                                                                    <  1:352335/287‑1 (MQ=255)`  
                                                                                                                          `cAATAGAAAAGAAACGTCCGGTTCTTAGTGTGGCAGGGACCCGCTTCCCGTTCGTCCGTCTGTTACTCAAACGAATCAAGGAGGTGTTCTGATGGGACTGTTAGATGGTGAAGCCTGGGAAAAAGAAAACCCGCCAGTACAAGAAACTGGGTGTATAGCTTGCTTAGAGAAAGATGACCGTTATCCACACACCTGTAACAAAGGAGCTAACGATATGACCGAACGTGAACAAGAGATGATCATTAAGTTGATAGACAATAATGAAGGTCGCCCAGATGATTTGAATGGCTGCGGTATtctc                                                                                                                                                                                    <  1:694746/301‑1 (MQ=255)`  
                                                                                                                           `tcgtgggctcggagatgtgtataagagacaggCAGTGACCCGCTTCCCGTTCGTCCGTCTGTTACTCAAACGAATCAAGGAGGTGTTCTGATGGGACTGTTAGATGGTGAAGCCTTGGAAAAAGAAAACCCGCCAGTACAAGCAACTGGGTGTATAGCTTGCTTAGAGAAAGATGACCGTTATCCACACACCTGTAACAAAGGAGCTAACGATATGACCGAACGTTAACAAGAGATGATCATTAAGTTGATAGACAATAATGAAGGTCGCCCAGATGATTTGAATGGCTGCGGTATTctct                                                                                                                                                                                   <  1:103092/271‑1 (MQ=255)`  
                                                                                                                                 `cAAGAAACGTCCGGTTCTTAGTGTGGCAGTGACCCGCTTCCCGTTCGTCCGTCTGTTACTCAAACGAATCAAGGAGGTGTTCTGATGGGACTGTTAGATGGTGAAGCCTGGGAAAAAGAAAACCCGCCAGTACAAGCAACTGGGTGTATAGCTTGCTTAGAGAAAGATGACCGTTATCCACACACCTGTAACAAAGGAGCTAACGATATGACCGAACGTGAACAAGAGATGATAATTAAGTTGATAGACAATAATGAAGGTCGCCCAGATGATTTGAATGGCTGCGGTATTCTCTGCTcct                                                                                                                                                                             >  1:174115/1‑300 (MQ=255)`  
                                                                                                                                    `gAAATGTCCGGTTCTTAGTGTGGCAGTGACCCGCTTCCCGTTCGTCCGTCTGTTACTCAAACGAATCAAGGAGGTGTTCTGATTGGACTGTTAGATGGTGAAGCCTGGGAAAAAGAAAACCCGCCAGTACAAGCAACTGGGTGTATAGCTTGCTTAGAGAAAGATGACCGTTATCCACACACCTGTAACAAAGGAGCTAACGATATGACCGAACGTGAACAAGAGATGATCATTAAGTTGATAGACAATAATGAAGGTCGCCCAGATGATTTGAATGGCTGCGGTATTCTCTGCTCCAATg                                                                                                                                                                          >  1:181967/1‑301 (MQ=255)`  
                                                                                                                                    `gAAACGTCCGGTTCTTAGTGTGGCAGTGACCCGCTTCCCGTTCGTCCGTCTGTTACTCAAACGAATCAAGGAGGTGTTCTGATGGGACTGTTAGATGGTGAAGCCTGGGAAAAAGAAAACCCGCCAGTACAAGCAACTGGGTGTATAGCTTGCTTAGAGAAAGATGACCGTTATCCACACACCTGTAACAAAGGAGCTAACGATATGACCGAACGTGAACAAGAGATGATCATTAAGTTGATAGACAATAATGAAGGTCGCCCAGATGATTTGAATGGCTGCGGTATTCTATGCTACAATg                                                                                                                                                                          <  1:366905/301‑1 (MQ=255)`  
                                                                                                                                       `gggtcgtgaatttgtataaggaacagcccGCTTCCCGTTCGTCCGTCTGTTACTCAAACGAATCAAGTAGGTGTTCTGATGGGACTGTTAGATGGTGAAGCCTGGGAAAAAGAAAACCCGCCAGTACAAGCAACTGGGTGTATAGCTTGCTTAGAGAAAGATGACCGTTATCCACACACCTGTAACAAAGGAGCTAACGATATGACCGAACGTGAACAAGAGATGATCATTAAGTTGATAGACAATAATGAAGGTCGCCCAGATGATTTGAATGGCTGCGGTATTCTCTGCTCCAATGTcc                                                                                                                                                                       <  1:516389/275‑1 (MQ=255)`  
                                                                                                                                        `cGTCCGGTTCTTAGTGTGGCAGTGACCCGCTTCCCGTTCGTCCGTCTGTTACTCAAACGAATCAAGGAGGTGTTCTGATGGGACTGTTAGATGGTTAAGCCTGGGAAAAAGAAAACCCGCCAGTACAAGCAACTGGGTGTATAGCTTGCTTAGAGAAAGATGACCGTTATCCACACACCTGTAACAAAGGAGCTAACGATATGACCGAACGTGAACAAGAGATGATCATTAAGTTGATAGACAATAATGAAGGTCGCCCAGATGATTTGAATGGCTGCGGTATTCTATGCTCCAATGTccc                                                                                                                                                                      <  1:168814/301‑1 (MQ=255)`  
                                                                                                                                              `gTTCTTAGTGTGGCAGTGACCCGCTTCCCGTTCGTCCGTCTGTTACTCAAACGAATCAAGGAGGTGTTCTGATGGGACTGTTAGATGGTGAAGCCTGGGAAAAAGAAAACCCGCCAGTACAAGCAACTGGGTGTATAGCTTGCTTAGAGAAAGATGACCGTTATCCACACACCTGTAACAAAGGAGCTAACGATATGACCGAACGTGAACAAGAGATGATCATTAAGTTGATAGACAATAATGAAGGTCGCCCAGATGATTTGAATGGCTGCGGTATTCTCTGCTCCAATGTCCCTTGCCa                                                                                                                                                                >  1:620282/1‑301 (MQ=255)`  
                                                                                                                                                  `ttAGTGTGGCAGTGACCCGCTTCCAGTTCGTCCGTCTGTTACTCAAACGAATCAAGGAGGTGTTCTGATGGGACTGTTAGATGGTGAAGCCTGGGAAAAAGAAAACCCGCCAGTACAAGCAACTGGGTGTATAGCTTGCTTAGAGAAAGATGACCGTTATCCACACACCTGTAACAAAGGAGCTAACGATATGACCGAACGTGAACAAGAGATGATCATTAAGTTGATAGACAATAATGAAGGTCGCCAAGATGATTTGAATGGCTGCGGTATTCTCTGCTCCAATGTCCCTTGCCACctc                                                                                                                                                            <  1:33526/301‑1 (MQ=255)`  
                                                                                                                                                  `ttAGGGTGGCAGTGACCCGCTTCCCGTTCGTCCGTCTGTTACTCAAACGAATCAAGGAGGTGTTCTGATGGGACTGTTAGATGGTGAAGCCTGGGAAAAAGAAAACCCGCCAGTACAAGCAACTGGGTGTATAGCTTGCTTAGAGAAAGATGACCGTTATCCACACACCTGTAACAAAGGAGCTAACGATATGACCGAACGTGAACAAGAGATGATCATTAAGTTGATATACAATAATGAAGGTCGCCCAGATGATTTGAATGGCTGCGGTATTCTCTGCTCCAATGTCCCTTGCCACctc                                                                                                                                                            <  1:395252/301‑1 (MQ=255)`  
                                                                                                                                                  `aatgtgtataagagacaggCTTCCCGTTCGTCCGTCTGTTACTCAAACGAATCAAGGAGGTGTTCTGATGGGACTGTTAGATGGTGAAGCCTGGGAAAAAGAAAACCCGCCAGTACAAGCAACTGGGTGTATAGCTTGCTTAGAGAAAGATGACCGTTATCCACACACCTGTAACAAAGGAGCTAACGATATGACCGAACGTGAACAAGAGATGATCATTAAGTTGATAGACAATAATGAAGGTCGCCCAGATGATTTGAATGGCTGCGGTATTCTCTGCTCCAATGTCCCTTGCCACctc                                                                                                                                                            <  1:9352/283‑1 (MQ=255)`  
                                                                                                                                                     `gtgtGGCAGTGACCCGCTTCCCGTTCGTCCGTTGGTTACTCAAACGAGTCAAGGAGGTGTGCTGATGGGACTGTTAGATAGTGAAGCCTAGGAAAAAGAAAACCCGCCAGTACAAGCAACTGGGTGTATAGCTTGCTTAGAGAAAGATGACCGTTATCCACACACCTGTAACAAAGGAGCTAACGATATGACCGAACGTGAACAAGAGATGATCATTAAGTTGATAGACAATAATGAAGGTCGCCCAGATGATTTAAATGGCTGCGGTATTCTCTGCACCAATGTCCCTTGCCACCTCTGc                                                                                                                                                         <  1:458122/301‑1 (MQ=255)`  
                                                                                                                                                                 `cccGCTCCCCTTTCGGCCGTCTGTTACTAAAACGAAGCAAGGAGGTGTTCTGATGGGACTGTTAGATGGTGAAGCCTGGGAAAAAGAAAACCCGCCAGTACAAGCAACTGGGTGTATAGCTTGCTTAGAGAAAGATGACCGTTATCCACACACCTGTAACAAAGGAGCTAACGATATGACCGAACGTGAACAAGAGATGATCATTAAGTTGATAGACAATAATGAAGGTCGCCCAGATGATTTGAATGGCTGCGGTATTCTCTGCTCCAATGTCCCTTGCCACCTCTGCCCCGCAAATAAc                                                                                                                                             <  1:748815/301‑1 (MQ=255)`  
                                                                                                                                                                     `cTTCCCGTTCGTCCGTCTGTTACTCAAACGAATCAAGGAGGTGTTCTGATGGGACTGTTAGATGGTGAAGCCTGGGAAAAAGAAAACCCGCCAGTACAAGCAACTGGGTGTATAGCTTGCTTAGAGAAAGATGACCGTTATCCACACACCTGTAACAAAGGAGCTAACGATATGACCGAACGTGAACAAGAGATGATCATTAAGTTGATAGACAATAATGAAGGTCGCCCAGATGATTTGAATGGCTGCGGTATTCTCTGCTCCAATGTCCCTTGCCACctcctgtctcttatacacatct                                                                                                                                         >  1:628921/1‑282 (MQ=255)`  
                                                                                                                                                                           `gTTCGTCCGTCTGTTACTCAAACGAATCAAGGAGGTGTTCTGATGGTACTGTTAGATGGTGAAGCCTGGGAAAAAGAAAACCCGCCAGTACAAGCAACTGGGTGGATAGCTTGCTTAGAGAAAGATGACCGTTATCCACACACCTGTAACAAAGGAGCTAACGATATGACCGAACGTGAACAAGAGATGATCATTAAGTTGATAGACAATAATGAAGGTCGCCCAGATGATTTGAATGGCTGCGGTATTCTCTGCTCCAATGTCCCTTTCCACCTCTGCCCCGCAAATAACGATCAAAAGa                                                                                                                                   <  1:804529/301‑1 (MQ=255)`  
                                                                                                                                                                                  `cgtcTGTTACTAAAACGAATCAAGGAGGTGTTCTGATGGGACTGTTAGATGGTGAAGCCTGGGAAAAAGAAAACCCGCCAGTACAAGCAACTGGGTGTATAGCTTGCTTAGAGAAAGATGACCGTTATCAACACACCTGTAACAAAGGAGCTAACGATATGACCGAACGTGAACAAGAGATGATCATTAAGTTGATAGACAATAATGAAGGTCGCCCAGATGATTTGAATGGCTGCGGTATTCTCTGCTCCAATGTCCCTTGCCACCTCTGCCCCGCAAATAACGATCAAAAGATAACCtt                                                                                                                            <  1:689749/301‑1 (MQ=255)`  
                                                                                                                                                                                        `ttACTCAAACGAATAAAGGAGGTGTTCTTATGGGACTGTGAGAGGGTTAAGCCTGGGAAAAAGAAAACCCGCCAGTACAAGAAACTGTGTGTATAGCTTGCTTAGAGAAAGATGACCGTTATCCACACACCTGTAACAAAGGAGCTAACGATATGACCGAACGTGAACAAGAGATGATCATTAAGTTGATAGACAATAATGAAGGTCGCCCAGATGATTTGAATGGCTGCGGTATTCTCTGCTCCAATGTCCCTTGCCACCTCTGCCCCGCAAATAACGATCAAAAGATAACCTTAGGTGa                                                                                                                      <  1:405942/301‑1 (MQ=255)`  
                                                                                                                                                                                                     `tCAAGGAGGTGTTCTGATGGGACTGTTAGATGGTGAAGCCTGTGAAAAAGAAAACCCGCCAGTACAAGCAACTGGGTGTATAGCTTGCTTAGAGAAAGATGACCGTTATCCACACACCTGTAACAAAGGAGCTAACGATATGACCGAACGTGAACAAGAGATGATCATTAAGTTGATAGACAATAATAAAGGTCGCCCAGATGATTTGAATGGCTGCGGTATTCTCTTCTCCAATGTCCCTTGCCACCTCTGCCCCGCAAATAACGATCAAAAGATAACCTTAGGTGAAATCCGAGCGATg                                                                                                         >  1:500165/1‑301 (MQ=255)`  
                                                                                                                                                                                                         `ggaggTGTTCTGATGGGACTGTTAGAGGGTGAAGCCTGGGAAAAAGAAAACCCGCCAGTACAAGCAACTGGTTGTATAGCTTGCTTAGAGAAAGATGACCGTTATCCACACACCTGTAACAAAGGAGCTAACGATATGACCGAACGTGAACAAGAGATGATCATTAAGTTGATAGACAATAATGAAGGTCGCCCAGATGATTTGAATGGCTGCGGTATTCTCTGCTCCAATGTCCCTTGCCACCTCTGCCCCGCAAATAACGATCAAAAGATAACCTTAGGTGAAATCCGAGCGATGGAcc                                                                                                     <  1:482663/301‑1 (MQ=255)`  
                                                                                                                                                                                                          `gaggTGTTCTGATGGGACTGTTAGATGGTGAAGCCTGGGAAAAAGAAAACCCGCCAGTACAAGCAACTGGGTGTATAGCTTGCTTAGAGAAAGATGACCGTTATCCACACACCTGTAACAAAGGAGCTAACGATATGACCGAACGTGAACAAGAGATGATCATTAAGTTGATAGACAATAATGAAGGTCGCCCAGATGATTTGAATGGCTGCGGTATTCTCTGCTCCAATGTCCCTTGCCACCTCTGCCCCGCAAATAACGATCAAAAGATAACCTTAGTTGAACTCCGAGCGATGGAccc                                                                                                    >  1:306956/1‑301 (MQ=255)`  
                                                                                                                                                                                                               `gtTCTGATGGGACTGTTAGATGGTGAAGCCTGGGAAAAAGAAAACCCTCCAGTACACGCAACTGGGTGTATAGCTTGCTTAGAGAAAGATGACCGTTATCCACACACCTGTAACAAAGGAGCTAACGATATGACCGAACGTGAACAATAGATGATCATTAAGTTGATAGACAATAATGAAGGTCGCCCAGATGATTTGAATGGCTGCGGTATTCTCTGCTCCAATGTCCCTTGCCACCTCTGCCCCGCAAATAACGATCAAAAGATAACCTTAGGTGAAATcctgtctcttatacacatct                                                                                               >  1:141640/1‑283 (MQ=255)`  
                                                                                                                                                                                                               `gtTCTGATGGGACTGTTAGATGGTGAAGCCTGGGAAAAAGAAAACCCGCCAGTACAAGCAACTGGGTGTATAGCTTGCTTAGAGAAAGATGACCGTTATCCACACACCTGTAACAAAGGAGCTAACGATATGACCGAACGTGAACAAGAGATGATCATTAAGTTGATAGACAATAATGAAGGTCGCCCAGATGATTTGAATGGCTGCGGTATTCTCTGCTCCAATGTCCCTTGCCACCTCTGCCCCGCAAATAACGATCAAAAGATAACCTTAGGTGAAATCCGAGCGATGGACCCACGTa                                                                                               >  1:266169/1‑301 (MQ=255)`  
                                                                                                                                                                                                               `gtTCTGATGGGACTGTTAGATGGTGAAGCCTGGGAAAAAGAAAACCAGCCAGTACAAGCAACTGGGTGTATAGCTTGCTTAGAGAAAGATGACCGTTATCCACACACCTGTAACAAAGGAGCTAACGATATGACCGAACGTGAACAAGAGATGATCATTAAGTTGATAGACAATAATGAAGGTCGCCCAGATGATTTGAATGGCTGCGGTATTCTCTGCTCCAATGTCCCTTGCCACCTCTGCCCCGCAAATAACGATCAAAAGATAACCTTAGGTGAAATCCGAGCGATGGACCCACGtc                                                                                               >  1:187446/1‑300 (MQ=255)`  
                                                                                                                                                                                                                     `gtgtataagagacagggTGAAGCCTGGGAAAAAGAAAACCCGCCAGTACAAGCAACTGGGTGTATAGCTTGCTTAGAGAAAGATGACCGTTATCCACACACCTGTAACAAAGGAGCTAACGATATGACCGAACGTGAACAAGAGATGATCATTAAGTTGATAGACAATAATGAAGGTCGCCCAGATGATTTGAATGGCTGCGGTATTCTCTGCTCCAATGTCCCTTGCCACCTCTGCCCCGCAAATAACGATCAAAAGATAACCTTAGGTGAAATCCGAGCGATGGACCCACGTAAACcac                                                                                         <  1:29774/286‑1 (MQ=255)`  
                                                                                                                                                                                                                          `cgtgggctcggagatgtgtataagagacagaaaCCCGCCAGTACAAGCAACTGGATGTATAGCTTGCTTAGAGAAAGATGACCGTTATCCACACACCTGTAACAAAGGAGCTAACGATATGACCGAACGTGAACAAGAGATGATCATTAAGTTGATAGACAATAATGAAGGTCGCCCAGATGATTTGAATGGCTGCGGTATTCTCTGCTCCAATGTCCCTTGCCACCTCTGCCCCGCAAATAACGATCAAAAGATAACCTTAGGTGAAATCCGAGCGATGGACCCACGTAAACCACATCTg                                                                                    <  1:541558/271‑1 (MQ=255)`  
                                                                                                                                                                                                                           `cTGTTAGATGGTGAAGCCTGGGAAAAAGAAAACCAGCCAGTACAAGCAACTGGGTGTATAGCTTGCTTAGAGAAAGATGACCGTTATCCACACACCTGTAACAAAGGAGCTAACGATATGACCGAACGTGAACAAGAGATGATCATTAAGTTGATAGACAATAATGAAGATCGCCCAGATGATTTGAATGGCTGCGGTATTCTCTGCTCCAATGTCCCTTGCCACCTCTGCCCCACAAATAACGATCAAAAGATAACCTTAGGTGAAATCCGAGCGATGGACCCACGTAAACCACATCTGa                                                                                   >  1:400442/1‑301 (MQ=255)`  
                                                                                                                                                                                                                             `tttAGATGGTGAAGCCTGGGAAAAAGAAAACCCGCCGGTACAAGCAACTGGGTGTATAGCTTGCTTAGAGAAAGATGACCGTTATCCACACACCTGTAACAAAGGAGCTAACGATATGACCGAACGTGAACAAGAGATGATCATTAAGTTGATAGACAATAATGAAGGTCGCCCAGATGATTTGAATGGCTGCGGTATTCTCTGCTCCAATGTCCCTTGCCACCTCTGCCCCGCAAAGAACGATCAAAAGATAACCTTAGGTGAAATCCGAGCGATGGACCCACGTAAACCACATCTGAAt                                                                                 <  1:340363/300‑1 (MQ=255)`  
                                                                                                                                                                                                                             `gTTAGATGGTGAAGCCTGGGAAAAAGAAAACCCGCCAGTACAAGCAACTGGGTGTATAGCTTGCTTAGAGAAAGATGACCGTTATCCACACACCTGTACCAAAGTAGCTAACGATATGACCGAACGTGAAGAAGCGATGATCATTAAGTTGATAGACAATAATGAAGCTCGCTCAGATCATTTGCATTGCTGCGGTATTCTCTGCTCCAATGTCCCTTGCCACCTCTGCCCCGCAAATAACGATGAAAAGATAACCTTAGGTGTAATCCGAGCGCTCGACCCACGTAAACCACATCTGAAt                                                                                 >  1:409225/1‑301 (MQ=255)`  
                                                                                                                                                                                                                             `gTTAGATGGTGAAGCCTGGGAAAAAGAAAACCCGCCAGTACAAGCAACTGGGTGTATAGCTTGCTTAGAGAAAGATGACCGTTATCCACACACCTGTAACAAAGGAGCTAACGATATGACCGAACGTGAACAAGAGATGATCATTAAGTTGATAGACAATAATGAAGGTCGCCCAGATGATTTGAATGGCTGCGGTATTCTCTGCTCCAATGTCCCTTGCCACCTCTGCCCCGCAAATAACGATCAAAAGATAACCTTAGGTGAAATCCGAGCGATGGACCCACGTAAACCACATCTGAAt                                                                                 >  1:535742/1‑301 (MQ=255)`  
                                                                                                                                                                                                                                  `aTGGTGAATCCTGGGAAAAAGAAAACCCGCCAGTACAAGCAACTGGGTGTATAGCTTGCTTAGAGAAAGATGACCGTTATCCACACACCTGTAACAAAGGAGCTAACGATATGACCGAACGTGAACAAGAGATGATCATTAAGTTGATAGACAAGAATGAAGGTCGCCCAGATGATTTGAATGGCTGCGGTATTCTCTGCTCCAATGTCCCTTGCCACCTCTGCCCCGCAAATAACGATCAAAAGATAACCTTAGGTGAAATCCGAGCGATGGACCCGCGTAAACCACATCTGAATAAAcc                                                                            <  1:120770/301‑1 (MQ=255)`  
                                                                                                                                                                                                                                              `gggAAAAAGAAAACCCGCCAGTACAAGCAACTGGGTGTATATCTTGCTTAGAGAAAGATGACCGTTATCCACACACCTGTAACAAAGGAGCTAACGATATGACCGAACGTGAACAAGAGATGATCATTAAGTTGATAGACAATAATGAAGGTCGCCCAGATGATTTGAATGGCTGCGGTATTCTCTGCTCCAATGTCCCTTGCCACCTCTGCCCAGAAAATAACGATCAAAAGATAACCTTAGGTGAAATCCGAGCGATGGACCCAAGTAAACAACATCTGAATAAACCTGAGGTAACTcc                                                                >  1:212498/1‑301 (MQ=255)`  
                                                                                                                                                                                                                                                 `aaaaaGAAAACCCGCCAGTACAAGCAACTGGGTGTATAGCTTGCTTAGAGAAAGATGACCGTTATCCACACACCTGTAACAAAGGAGCTAACGATATGACCGAACGTGAACAAGAGATGATCATTAAGTTGATAGACAATAATGAAGGTCGCCCAGATGATTTGAATGGCTGCTGTATTCTCTGCTCCAATGTCCCTTGCCACCTCTGCCCCGCAAATAACGATCAAAAGATAACCTTAGGTGAAATCCGAGCGATGGACCCACGTAAACCACATCTGAATAAACCTGAGGTAACTCCTAc                                                             <  1:791388/301‑1 (MQ=255)`  
                                                                                                                                                                                                                                                   `gtgggctcggagatgtgtataagagacaggTGTATAGCTTGCTTAGAGAAAGATGACCGTTATCCACACACCTGTAACAAAGGAGCTAACGATATGACCGAACGTGAACAAGAGATGATCATTAAGTTGATAGACAATAATGAAGGTCGCCCAGATGATTTGAATGGCTGCGGTATTCTCTGCTCCAATGTCCCTTGCCACCTCTGCCCAGCAAATAACGATCAAAAGATAACCTTAGGTGAAATCCGAGCGATGGACCCACGTAAACCACATCTGAATAAACCTGAGGTAACTCCTACAg                                                           <  1:79866/273‑1 (MQ=255)`  
                                                                                                                                                                                                                                                   `gggctcggagatgtgtataagagacaggggTGTATAGCTTGCTTAGAGAAAGATGACCGTTATCCACACACCTGTAACAAAGGAGCTAACGATATGACCGAACGTGAACAAGAGATGATCATTAAGTTGATAGACAATAATGAAGGTCGCCCAGATGATTTGAATGGCTGCGGTATTCTCTGCTCCAATGTCCCTTGCCACCTCTGCCCCACAAATAACGATCAAAAGATAACCTTAGGTGAAATCCGAGCGATGGACACACGTAAACCACATCTGAATAAACCTGAGGTAACTCCTACAg                                                           <  1:529711/274‑1 (MQ=255)`  
                                                                                                                                                                                                                                                   `aaaTAAAACCCGCCAGTACAAGCAACTGGGTGTATAGCTTGCTTAGAGAAAGATGACCGTTATCCACACACCTGTAACAAAGGAGCTAACTATATGACCGAACGTGAACAAGAGATGATCATTAAGTTGATAGACAATAATGAAGGTCGCCCAGATGATTTGAATGGCTGCGGTATTCTCTGCTCCAATGTCCCTTGCCACCTCTGCCCCGCAAATAACGATCAAAAGATAACCTTAGGTGAAATCCGAGCGATTGACCCACGTAAACCACATCTGAATAAACCTGAGGTAACTCCTACAg                                                           <  1:442069/301‑1 (MQ=255)`  
                                                                                                                                                                                                                                                    `aaGAAAACCCGCCAGTACAAGCAACTGGGTGTATAGCTTGCTTAGAGAAAGATGACCGTTATCCACACACCTGTAACAAAGGAGCTAACGATATGACCGAACGTGAACAAGAGATGATCATTAAGTTGATAGACAATAATGAAGGTCGCCCAGATGATTTGAATGGCTGCGGTATTCTCTGCTCAAATGTCCCTTGCCACCTCTGCCCCGCAAATAACGATCAAAAGATAACCTTAGGTGAAATACGAGCGATGGACCCACGTAACCCACATCTGAATAAACCTGAGGTAACTCATACAGa                                                          >  1:215297/1‑301 (MQ=255)`  
                                                                                                                                                                                                                                                                  `gTACAAGCAACTGGTTGTATAGCTTGCTTAGAGAAAGATGACCGTTATCCACACACCTGTAACAAAGGATCTCACGATATGACCGAACGTGAACAAGAGATGATCATTAAGTTGATAGACAATAATGAAGGTCGCCCAGATGATTTGAATGGCTGCGGTATTCTCTGCTCCAATGTCCCTTGCCACCTCTGCCCCGCAAATAACGATCAAAAGATAACCTTAGGTGAAATCCGAGCGATGGACCCACGTAAACCACATCTGAATAAACCTGAGGTAACTCCTACAGATGACCAGCCTTCCg                                            >  1:86005/1‑301 (MQ=255)`  
                                                                                                                                                                                                                                                                   `tACAAGCAACTGGGTGTATAGCTTGCTTAGAGAAAGATGACCGTTATCCACACACCTGTAACAAAGGAGCTAACGATATGACCGAACGTGAACAAGAGATGATCATTAAGTTGATAGACAATAATGAAGGTCGCCCAGATGATTTGAATGGCTGCGGTATTCTCTGCTCCAATGTCCCTTGCCACCTATGCCCCGCAAAGAACGATCAAAAGATAACCTTAGGTGAAATCCGAGCGATGGACCAACGTAAACCACATCTGAATAAACCTGAGGTAACTCCTACAGATGACCAGCCTTCCga                                           <  1:109343/301‑2 (MQ=255)`  
                                                                                                                                                                                                                                                                         `cAACTGGGTGTATAGCTTGCTTAGAGAAAGATTACCGTTATCCACACACCTGTAACAAAGGAGCTAACGATATGACCGAACGTGAACAAGAGATGATCATTAAGTTGATAGACAATAATGAAGGTCGCCCAGATGATTTGAATGGCTGCGGTATTCTCTGCTCCAATGTCCCTTGCCACCTCTGCCCCGCAAATAACGATCAAAAGATAACCTTAGGTGAAATCCGAGCGATGGACCCACGTAAACCACATCTGAATAAACCTGAGGTAACTCCTACAGATGACCAGCCTTCCGCTGAGAc                                     <  1:173218/301‑1 (MQ=255)`  
                                                                                                                                                                                                                                                                         `cAACTGGGTGTATAGCTTGCTTAGAGAAAGATGACAGTTATCCACACACCTGTAACAAAGGAGCTAACGATATTACCGAACGTGAACAAGAGATGATCATTAAGTTGATAGACAATAATGAAGGTCGCCCAGATGATTAGAATGGCTGCGGTATTCTCTGCTCCAATGTCCCCTGCCACCTCTGCCCCGCAAATAACGATCAAAAGATAAACTTAGGTGAAATCCGAGCGATGGACCCACGTAAACCACATCTGAATAAACCTGAGGTAACTCCTACAGATGACCAGCCATCCGCTgagaa                                     >  1:237229/1‑300 (MQ=255)`  
                                                                                                                                                                                                                                                                              `gggTGTATAGCTTGCTTAGAGAAAGATGACCGTTATCCACACACCTGTAACAAAGGAGCTAACGATATGACCGAACGTGAACAAGAGATGATCATTAAGTTGATAGACAATAATGAAGGTCGCCCAGATGATTTGAATGGCTGCGGTATTCTCTGCTCCAATGTCCCTTGCCACCTCTGCCCCGCAAATAACGATCAAAAGATAACCTTAGGTGAAATCCGAGCGATGGACCCACGTAAACCACATCTGAATAAACCTGAGGTAACTCCTACAGATGACCAGCCTTCTGCTGAGACAATCg                                >  1:140178/1‑301 (MQ=255)`  
                                                                                                                                                                                                                                                                                   `tataGCTTGCTTAGAGAAAGATGACCGTTATCCACACACCTGTAACAAAGGAGCTAACGATATGACCGAACGTGAACAAGAGATGATCATTAAGTTGATAGACAATAATGAAGGTCGCCCAGATGATTTGAATGGCTGCGGTATTCTCTGCTCCAATGTCCCTTGCCACCTCTGCCCCGCAAATAACGATCAAAAGATAACCTTAGGTGAAATCCGAGCGATGGACCAACGTAAACCACATCTGAATAAACCTGAGGTAACTCCTACAGATGACCAGCCTTCCGCTGAGACAATCGAAGgt                           <  1:37670/301‑1 (MQ=255)`  
                                                                                                                                                                                                                                                                                    `ataGCTTGCTTAGAGAAAGATGACCGTTATCCACACACCTGTAACAAAGGAGCTAACGATATGACCGAACGTGAACAAGAGATGATCATTAAGTTGATAGACAATAATGAAGGTCGCCCAGATGATTTGAATGGCTGCGGTATTCTCTGCTACAATGTCCCTTGCCACCTCTGCCCCGCAAATAACGATCAAAAGATAACCTTAGGTGAAATCCGAGCGATGGACCCACGTAAACCACATCTGAATAAACCTGAGGTAACTCCTACAGCTgtctcttatacacatctccgagcccacgaga                          >  1:602383/1‑271 (MQ=255)`  
                                                                                                                                                                                                                                                                                        `cttgcttAGAGAAAGATGACCGTTATCCACACACCTGTAACAAAGGAGCTAACGATATGACCGAACGTGAACAAGAGATGATCATTAAGTTGATAGACAATAATGAAGGTCGCCCAGATGATTTGAATGGCTGCGGTATTCTCTGCTCCAATGTCCCTTGCCACCTCTGCCCCGCAAATAACGATCAAAAGATAACCTTAGGTGAAATCTGAGCGATGGACCCACGTAAACCACATCTGAATAAACCTGAGGTAACTCCTACAGATGACCAGCCTTCCGCTGAGACAATCGAAGGTGTCAc                      >  1:553942/1‑301 (MQ=255)`  
                                                                                                                                                                                                                                                                                              `tAGAGAAAGATGACCGTTATCCACACACCTGTAACAAAGGAGCTAACGATATGACCGAACGTGAACAAGAGATGATCATTAAGTTGATAGACAATAATGAAGGTCGCCCAGATGATTTGAATGGCTGCGGTATTCTCTGCTCCAATGTCCCTTGCCACTTCTGCCCCGCAAATAACGATCAAAAGATAACCTTAGGTGAAATCCGAGCGATGGACCCACGTAAACCACATCTGAATAAACCTGAGGTAACTCCTACAGATGACCAGCCTTCCGCTTAGACAATCGAAGGTGTCAATAAGcc                >  1:432991/1‑301 (MQ=255)`  
                                                                                                                                                                                                                                                                                                        `tGACCGTTATCCACACACCTGTAACAAAGTAGCTAACGATATGACCGAACCTGAACAAGAGATGATCATTAAGTTGATAGACAATCATGAAGGTCGCCCAGATGATTTGAATGGCTGCGGTATTCTCTGCTCCAATGTCCCTTGCCACCTCTGCCCCGCAAATAACGATCAAAAGATAACCTTAGGTGAAATCCGAGCGATGGACCCACGTAAACCACATCTGAATAAACCTGAGGTAACTCCTACAGATGACCAGCCTTCCGCTGAGACAATCGAAGGTGTCACTAAGCCTTCCAACTAc      <  1:147990/301‑1 (MQ=255)`  
                                                                                                                                                                                                                                                                                                          `aCCGTTATCCACACACCTGTAACAAAGGAGCTAACGATATGACCGAACGTGAACAAGAGATGATCATTAAGTTGATAGACAATAATGAAGGTCGCCCAGATGATTTGAATGGCTGCGGTATTCTCTGCTCCAATGTCCCTTGCCACCTCTGCCCCGCAAATAACGATCAAAAGATAACCTTATGTGAAATCCGAGCGATGGACCCACGTAAACCACATCTGAATAAACCTGAGGTAACTCCTACAGATGACCAGCCTTCCGCTGAGACAATCGAAGGTGTCACTAAGCCTTCCCACTACAt    >  1:597447/1‑301 (MQ=255)`  
                                                                                                                                                                                                                                                                                                            `cGTTATCCACACACCTGTAACAAAGGAGCTAACGATATGACCGAACGTGAACAAGAGATGATCATTAAGTTGATAGACAATAAAGAAGGTCGCCCAGATGATTTGAATGGCTGCGGTATTCTCTGATCCAATGTCCCTTGCCACCTCTGCCCCGCAAATAACGATCAAAAGATAACCTTAGGTGAAATCCGAGCGATGGACCCACGTAAACCACATCTGAATAAACCTGAGGTAACTCCTACAGATGACCAGCCTTCCGCTGAGACAATCGAAGGTGTCACTAAGCCTTCACACTACATGc  <  1:727643/301‑1 (MQ=255)`  

                                                                                                                                                                                                                                                                                                            `|`                                                                                                                                                                                                                                                                                                              
`CCGTGCTGACCGTTCAATCGTATGTGCGAGCGAGCGCCGAGCTAAGATACCTCTTATTGGTAACACAGTTCCTTTGGCACCGAGCGTCCACATCATTATCACCCGTGGTGACTTTGAGAAAGCAATAGACAAGAAACGTCCGGTTCTTAGTGTGGCAGTGACCCGCTTCCCGTTCGTCCGTCTGTTACTCAAACGAATCAAGGAGGTGTTCTGATGGGACTGTTAGATGGTGAAGCCTGGGAAAAAGAAAACCCGCCAGTACAAGCAACTGGGTGTATAGCTTGCTTAGAGAAAGATGACCGTTATCCACACACCTGTAACAAAGGAGCTAACGATATGACCGAACGTGAACAAGAGATGATCATTAAGTTGATAGACAATAATGAAGGTCGCCCAGATGATTTGAATGGCTGCGGTATTCTCTGCTCCAATGTCCCTTGCCACCTCTGCCCCGCAAATAACGATCAAAAGATAACCTTAGGTGAAATCCGAGCGATGGACCCACGTAAACCACATCTGAATAAACCTGAGGTAACTCCTACAGATGACCAGCCTTCCGCTGAGACAATCGAAGGTGTCACTAAGCCTTCCCACTACATGC  >  NC_001604.1/7953‑8553`

  

Alignment Legend

Aligned base mismatch/match (shaded by quality score): `ATCG/ATCG` `< 3 ≤` `ATCG/ATCG` `< 10 ≤` `ATCG/ATCG` `< 22 ≤` `ATCG/ATCG` `< 34 ≤` `ATCG/ATCG` `< 38 ≤` `ATCG/ATCG`

Unaligned base: `atcg`    Masked matching base: `atcg`    Alignment gap: `‑`    Deleted base: `‑`

Reads not counted as support for junction

read\_name Not counted due to insufficient overlap past the breakpoint.

read\_name Not counted due to not crossing MOB target site duplication.
